# Invasive Fungal Infection by *Scedosporium apiospermum* with Cerebral Involvement in a Pediatric Patient Affected by Chronic Granulomatous Disease After Hematopoietic Cell Transplant

**DOI:** 10.3390/jof11040270

**Published:** 2025-04-01

**Authors:** Chiara Garonzi, Matteo Chinello, Giulia Caddeo, Elisa Bonetti, Maria Pia Esposto, Vincenza Pezzella, Virginia Vitale, Ada Zaccaron, Annarita Sorrentino, Davide Gibellini, Simone Cesaro

**Affiliations:** 1 Pediatric Hematology Oncology, Department of Mother and Child, Azienda Ospedaliera Universitaria Integrata Verona, 37126 Verona, Italy; matteo.chinello@aovr.veneto.it (M.C.); giulia.caddeo@aovr.veneto.it (G.C.); elisa.bonetti2@aovr.veneto.it (E.B.); mariapia.esposto@aovr.veneto.it (M.P.E.); vincenza.pezzella@aovr.veneto.it (V.P.); virginia.vitale@aovr.veneto.it (V.V.); ada.zaccaron@aovr.veneto.it (A.Z.); simone.cesaro@aovr.veneto.it (S.C.); 2 Microbiology Unit, Azienda Ospedaliera Universitaria Integrata Verona, 37126 Verona, Italy; annarita.sorrentino@aovr.veneto.it; 3 Department of Diagnostic and Public Health, Microbiology Section, University of Verona, 37134 Verona, Italy; davide.gibellini@univr.it

**Keywords:** *Scedosporium apiospermum* infection, mycoses, allogeneic hematopoietic cell transplantation, chronic granulomatous disease

## Abstract

A 5-year-old boy affected by chronic granulomatous disease (CGD) underwent two allogeneic hematopoietic cell transplants (HCT) from the same unrelated donor. The first HCT was complicated by prolonged fever and primary graft failure. While fully aplastic, the patient developed a disseminated infection by *Scedosporium apiospermum* involving the knee and parasternal skin (day +34 and +40 post-HCT). The patient was treated with voriconazole and granulocyte transfusions followed by a second HCT 80 days after the first HCT. At day +105, the patient developed fever, headache, and altered level of consciousness associated with multiple bilateral cerebral abscesses at magnetic resonance imaging. The serum B-D-glucan test was positive. Micafungin was added to voriconazole. Despite an initial clinical improvement, the patient developed hydrocephalus. *Scedosporium apiospermum* was cultured from cerebrospinal fluid. Liposomal amphotericin B, instead of micafungin, was combined with voriconazole as salvage therapy. Unfortunately, the patient developed uncal herniation and died at day +193 from HCT. This case shows that the prognosis of scedosporiosis remains poor despite adequate antifungal treatment. Noteworthy, the B-D-Glucan test is confirmed useful as a non-invasive marker for early diagnosis and may help the differential diagnosis of mycoses.

## 1. Introduction

Invasive fungal infections have been emerging globally in the last few decades, leading to elevated rates of morbidity and mortality [1,2]. Although most invasive infections by mold pathogens can be attributed to *Aspergillus* spp., other molds, such as *Mucorales* spp., *Scedosporium* spp., *Lomentospora prolificans*, and *Fusarium* spp., are increasingly reported, accounting for 10–25% of all invasive mold disease in patients with hematological malignancy or after hematopoietic cell transplant (HCT) [3]. *Scedosporium* spp. are saprophytes that can be ubiquitously found in the environment, especially in soil, sewage, and polluted waters [4]. Species from the *Scedosporium* (*S.*) species complex, such as *S. apiospermum* and *S. boydii*, and *S. aurantiacum*, are the most common [3]. The incidence of scedosporiosis increased through the years, with 0.82 vs. 1.33 cases per 100,000 patient-inpatient days from 1993 to 2005, respectively [5]. In immunocompromised patients, the infection is frequently disseminated with high mortality (46%) [6]. Since *S.* spp. are intrinsically resistant to many antifungals, an early diagnosis is fundamental to starting the proper therapy [7].

We report a clinical case of scedosporiosis with cerebral involvement in a 5-year-old child who underwent two HCTs for chronic granulomatosis disease (CGD).

## 2. Case Presentation

A 5-year-old boy was diagnosed with X-linked CGD by genetic testing (a pathogenic variant of the CYBB gene) after a history characterized by multiple lymphadenopathies and recurrent mycobacterial and Gram-positive infections (*S. aureus*) with lung involvement. At the diagnosis of CGD, the patient was receiving anti-tubercular therapy because of a previous *Mycobacterium tuberculosis* complex positivity obtained from a lymph node drainage. After the diagnosis of CGD, the patient was started on cotrimoxazole prophylaxis for *Pneumocytis jirovecii* pneumonia and antifungal prophylaxis with liposomal amphotericin B (3 mg/kg/dose, twice a week; then 5 mg/kg/dose, once a week). This last was preferred to azole antifungals to prevent drug-to-drug interactions with the concomitant anti-tubercular therapy (isoniazid, rifampin, ethambutol, and pyrazinamide).

After 13 months from CGD diagnosis, the patient underwent allogeneic HCT from an unrelated donor (male donor, 45 years, recipient/donor HLA A, B, C, DR match: 7/8). The conditioning regimen and the graft-versus-host disease (GVHD) prophylaxis are reported in Table 1. The patient received a bone marrow graft containing 5 × 10^6^/kg CD34+ cells. The HCT was complicated by prolonged fever (from day +3 to +47 post-HCT), treated empirically with broad-spectrum antibiotics (meropenem, amikacin, and teicoplanin; then switched to ceftazidime/avibactam and Fosfomycin; this last one changed to clindamycin; with transient de-escalation to piperacillin/tazobactam during a phase of clinical improvement) and liposomal amphotericin B (3 mg/kg/dose every 24 h), primary graft failure (GF, day +41), and sepsis by Bacillus cereus (day +69, treated with meropenem and vancomycin). While fully aplastic, the patient developed abscesses in the knee joint and parasternal site (day +34 and +40, respectively). The drainage of the parasternal abscess (day +60) resulted in the isolation of *Scedosporium apiospermum*. Concomitant serum B-D-glucan tests (Toxinometer MT-6500, FUJIFILM Wako Pure Chemical Corporation) resulted in negative (<4 pg/mL). The patient was treated empirically with voriconazole (load: 9 mg/kg/dose every 12 h for two doses, then 8 mg/kg/dose every 12 h) and five granulocyte transfusions (GTX) using the father as a donor. Therapeutic drug monitoring (TDM) of voriconazole was performed (target trough level: 1–5 μg/mL). At the in vitro susceptibility test (broth dilution, Thermo Scientific (Waltham, MA, USA), Sensititre ^TM^ YeastOne ITAMYUCC, manufactured by TREK Diagnostic Systems, Units 17-19 Birches Industrial Estate, East Grinstead, West Sussex, Crawley, UK), a MIC of 0.25 resulted in voriconazole (Table 2).

A second HCT was performed at +80 days from the first HCT, using the same donor. The conditioning regimen and the GVHD prophylaxis are reported in Table 1. The patient received a bone marrow graft infusion containing 3.65 × 10^6^/kg CD34+ cells. This HCT was complicated by hepatic veno-occlusive disease, cytomegalovirus infection, respiratory syncytial virus, influenza infections, recurrent pericardial effusion (microbiologically negative, including fungal cultures), and grade III skin GVHD, treated with intravenous steroids and ruxolitinib. Nonetheless, with the gradual myeloid recovery, fungal abscesses in the knee joint and parasternal progressively improved. Neutrophil engraftment occurred at day + 29 post-second HCT. The patient was discharged from the hospital on day +88 from the second HCT, still on oral voriconazole therapy (9 mg/kg/dose every 12 h). Due to poor graft function, the patient needed occasional platelets, red blood cell transfusions, and granulocyte-colony stimulating factor administrations.

At day +105, the child was admitted to the hospital due to fever, headache, and altered level of consciousness. At that time, GVHD prophylaxis was with ruxolitinib twice a week and prednisone (on tapering). Antifungal therapy had been switched to Amphotericin B (3 mg/kg/dose, twice a week) about two weeks before, due to hepatic toxicity attributed to voriconazole. Blood counts were WBC 1.57 × 10^9^/L (PMN 1 × 10^9^/L) and platelets 16 × 10^9^/L. Magnetic resonance imaging revealed multiple bilateral cerebral abscesses in the cortical-subcortical regions. The serum B-D-glucan test was positive (9.05 pg/mL, positive if >7), while serum galactomannan resulted in negative. In the hypothesis of a possible mycosis [8], the patient started voriconazole (load: 9 mg/kg/dose every 12 h for two doses, then 8 mg/kg/dose every 12 h), then micafungin was added (2 mg/kg/dose every 24 h), and immunosuppression was reduced. The analysis of cerebrospinal fluid obtained from a spinal tap showed low glucose levels, elevated proteins, and white blood cell counts. Microbiological investigations (including fungal culture) on cerebrospinal fluid resulted in negative, except for the B-D-glucan test (7.35 pg/mL, at day + 114). At the following cerebrospinal fluid examination (day +126), fungal culture resulted in negative, as well as the B-D-glucan. Therapeutic drug monitoring of voriconazole was periodically performed to optimize the drug dose, with a target trough level between 1 and 5 μg/mL. Noteworthy, from day +118 to +139, voriconazole was temporarily switched to posaconazole (6.5 mg/kg/day) due to gastrointestinal toxicity (vomiting). Meanwhile, chimerism analysis, performed both on peripheral blood and bone marrow, showed full donor chimerism, and neutrophil superoxide production at the dihydrorhodamine oxidation assay resulted in normal.

**Figure 1:**
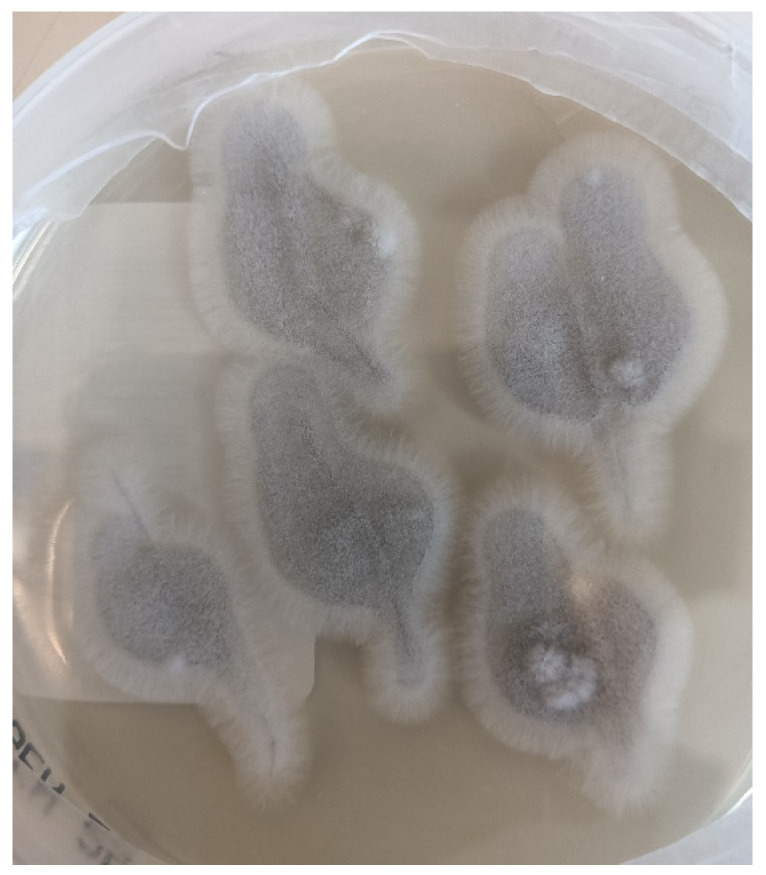
*Scedosporium apiospermum* on Sabouraud Dextrose agar culture (photography of culture plate, plate diameter: 8.5 cm).

**Figure 2:**
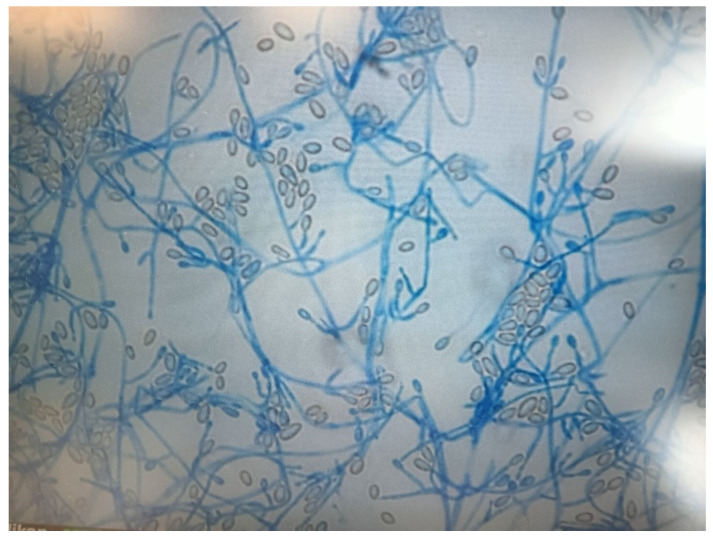
*Scedosporium apiospermum* at microscopic examination (lactophenol cotton blue staining; microscope objective 25/0.63, eyepiece GF 12.5×/20).

**Figure 3:**
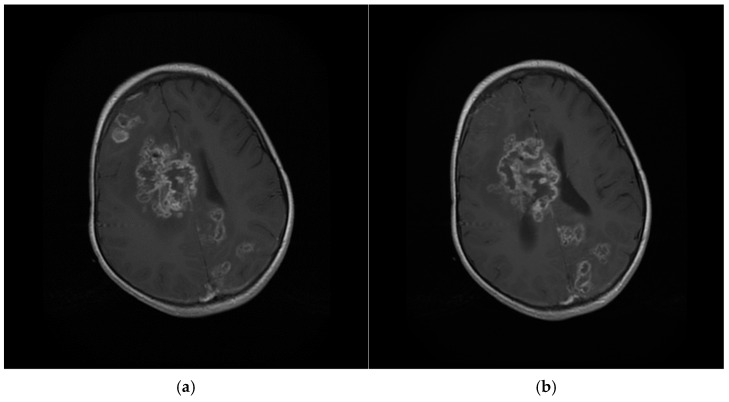
Brain MRI ((**a**,**b**): different slices), day + 183 post-2nd HCT.

Despite an initial clinical neurological improvement, the patient developed pericardial effusion with hemodynamic instability that required a cardiosurgical intervention of pericardiotomy, pleuro-pericardial window, and positioning of drainage (day +140). The pericardial fluid was negative at microbiological culture (including fungal cultures). In the following days, the child developed a progressive neurological impairment associated with triventricular hydrocephalus that required the positioning of external drainage at day + 172. This time, *S. apiospermum* was identified by the culture of cerebrospinal fluid (Figure 1 and Figure 2) and confirmed at the Matrix-Assisted Laser Desorption Ionization-Time of Flight Mass Spectrometry (Vitek^®^ MS, bioMérieux, Marcy-l’Étoile, France), along with high B-D-glucan levels in cerebrospinal fluid (173.3 pg/mL). Considering the worsening of *S. apiospermum* infection despite long-lasting voriconazole therapy, liposomal amphotericin B (5 mg/kg/dose every 24 h) was empirically added as salvage therapy instead of micafungin. Antifungal susceptibility testing is reported in Table 2. Figure 3a,b represent the MRI at day + 183. Unfortunately, in the following days, the patient developed progressive cerebral edema, uncal herniation, coma, and died on day + 193. Noteworthy, fungal susceptibility testing for antifungal combinations was performed at a national reference center. For the determination of the interaction, a microdilution checkerboard technique based on the Clinical and Laboratory Standards Institute (CLSI) reference method for antifungal susceptibility testing was used [9], showing that the more efficient combination in vitro seemed to be voriconazole *plus* micafungin. Still, unfortunately, this result arrived only post-mortem.

## 3. Discussion

We reported the case of a fatal fungal infection with CNS involvement by *S. apiospermum*. Few studies reported cases of scedosporiosis in HCT recipients. In 2005, Husain et al. analyzed 23 cases of S. spp. infections (including 9 *Lomentospora prolificans* infections, previously named *S. prolificans*) in HCT recipients. Overall, 69% of infections were disseminated with a mortality rate of 68%. Antifungal therapy with voriconazole was associated with a trend of better survival, compared with amphotericin B [10]. A fatal CNS infection by *S. apiospermum* after HCT was described in 2002 and treated with amphotericin B (voriconazole not available) [11]. A rhinoencephalitis due to *S. apiospermum*, 8 months after allogeneic-HCT, was reported in a 17-year-old boy with concomitant Rhizomucor pneumonia. The patient was treated with posaconazole and liposomal amphotericin B, but he had no response and, subsequently, experienced a fatal outcome [12]. Moreover, a 65-year-old HCT recipient with acute myeloid leukemia (AML) developed a disseminated scedosporiosis involving the lungs and CNS, probably from a foot source (toe wound). A combination therapy with voriconazole and liposomal amphotericin B successfully treated the infection, despite physical and neurological sequelae that took a long recovery [13]. Interestingly, a 12-year-old boy with CGD and pericardial infection by *S. apiospermum*, initially treated with voriconazole and GTX, underwent HCT to control the persistent and life-threatening infection. Radiologic investigations documented a post-transplant resolution of the S. spp. infection [14]. Finally, ocular complications have also been reported after HCT, including a case of chorioretinitis associated with disseminated scedosporiosis with osteomyelitis, endocarditis, and brain abscess, successfully treated with voriconazole, amphotericin B, and surgery, and a case of endophthalmitis after a second allogeneic HCT for AML [15,16]. Studies and case reports of scedosporiosis are summarized in Table 3.

In this case, we reported several cofactors predisposing to the mycosis. First, the long-lasting defective function of granulocytes was due to the underlying disease that resulted in the defective bactericidal and fungicidal activity [17]. Moreover, the prolonged aplasia that had followed HCT, due to the primary GF, and the deep post-transplant immunosuppressive therapy used as GVHD prophylaxis played a role in the development and dissemination of the infection. Finally, the poor graft function might have contributed to the scarce control of the disease.

HCT is a potentially definitive treatment for CGD. A recent multicenter study demonstrated that allogeneic HCT could reduce the burden of the disease, improving growth and nutrition, resolving infections and inflammatory disease, and reducing rates of antimicrobial prophylaxis or corticosteroid use. The study reported a 3-year overall survival of 82% and event-free survival of 69% [18]. However, in patients with reduced performance status, high inflammation before HCT, and HLA-mismatched donors, there were augmented rates of both GF and chronic GVHD [18,19].

**Figure 4:**
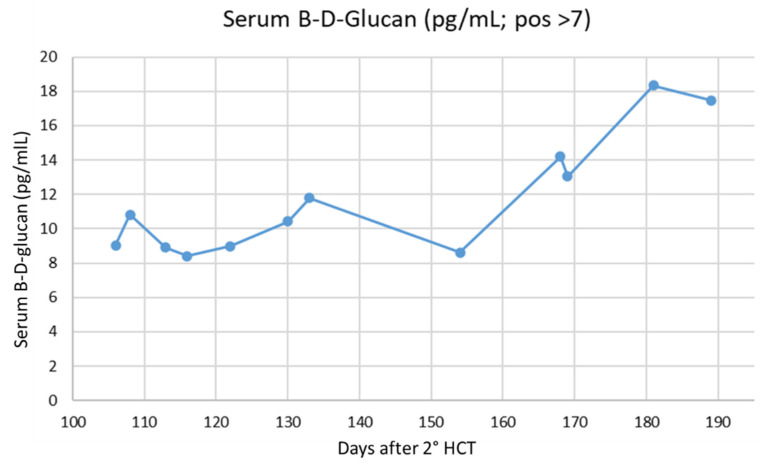
Serum B-D-glucan test results (Wako turbidimetric assay).

We also found that abnormal results of the B-D-glucan test were associated with the occurrence and worsening of scedosporiosis (serum B-D-glucan levels are reported in Figure 4). Indeed, the B-D-glucan test was already positive at the time of the first central nervous system symptoms (9.05 pg/mL, positive if >7). This is consistent with a recent meta-analysis that reported a sensitivity of the B-D-glucan test for the diagnosis of scedosporiosis of 81.5%. The meta-analysis showed that positive results of B-D-glucan preceded the diagnosis by conventional methods (i.e., culture, histopathology) in 94% of cases. Finally, the combined use of the B-D-glucan test and galactomannan may help differentiate between invasive mold infections [20]. The B-D-glucan test in cerebrospinal fluid was also helpful, being the only positivity detected during the first examinations (Table 4).

Despite the parasternal and knee joint abscesses by *Scedosporium* improving with voriconazole therapy, the patient died from the cerebral localization of the mycosis. Unfortunately, we do not know if cerebral involvement was present since the initial diagnosis of scedosporiosis, though neurologically asymptomatic, because neither a head MRI nor computed tomography was performed.

The recommended treatment for scedosporiosis is voriconazole, which showed better outcomes compared with amphotericin B. Therefore, the use of amphotericin B monotherapy is discouraged whenever voriconazole is available [3,7,21]. Alternatives are posaconazole and voriconazole-based antifungal combinations, including voriconazole plus terbinafine or echinocandin or amphotericin B [3,21]. Instead, neither isavuconazole nor itraconazole is currently recommended [7]. Salvage therapy can be necessary in case of refractory infection or toxicity/intolerance, using a voriconazole-based antifungal combination or posaconazole, in case of intolerance or resistance to VCZ [7]. Due to the pharmacokinetic unpredictability and the inter-patient variability, the TDM is recommended during azole use to guide the dosing and avoid treatment failure and toxicities [3]. In our case, the antifungal susceptibility testing confirmed the sensitivity of voriconazole. However, due to suspected drug toxicities, we suspended voriconazole twice. In the first case for hepatic toxicity, since parasternal and knee joint abscesses were clinically and radiologically improved, voriconazole was temporarily switched to amphotericin B. In the second case, voriconazole was temporarily switched to posaconazole due to gastrointestinal toxicity. Moreover, we hypothesize that the non-response to antifungals might be related to profound and prolonged immunosuppression and insufficient concentration in the cerebral lesions.

New promising antifungal drugs are under investigation. For scedosporiosis, olorofilm and fosmanogepix have shown activity, both in vitro and in animal studies [7]. Olorofilm belongs to the new orotomide class that inhibits the fungal dihydroorotate dehydrogenase responsible for fungal pyrimidine and DNA synthesis. Fosmanogepix is a pro-drug of manogepix, a glycosylphosphatidylinositol anchor inhibitor [3]. Therefore, we expect to have new weapons against fungal infections in the near future.

## 4. Conclusions

Invasive fungal infections by *Scedosporium* spp. are an emerging global issue due to the high morbidity and mortality rates, especially in HCT recipients. In this case, we conclude that the long-lasting defective function of PMN due to CGD, the prolonged aplasia due to primary graft failure, and deep post-transplant immunosuppression have been significant factors in the occurrence of this rare mycosis. What we have learned is that the screening of CNS localization by MRI in severely immunocompromised patients with *Scedosporium* infections would be useful to define the extension of the disease, even though neurologically asymptomatic. Moreover, we found that the B-D-glucan test was useful for diagnosing and monitoring invasive scedosporiosis. Finally, the introduction of new antifungal drugs in the next years may improve the prognosis of this severe infection.

## Figures and Tables

**Table 1:** Conditioning regimen and GVHD prophylaxis used in the HCT.

	Drug, Dose	Timing
First HCT		
Conditioning regimen	Busulfan, 1 mg/kg/dose for 16 doses over 5 days	From day −11 to day −7
	Fludarabine, 40 mg/m^2^/day for 4 days	From day −2 to −5
	ATG Genzyme^®^, 1.5 mg/kg/dose for 6 doses in total	From day −7 to day −3
GVHD prophylaxis	Cyclophosphamide, 50 mg/kg	Days +4 and +5 post-HCT
	Cyclosporine, 3 mg/kg/day	From day +5
	Mycophenolate mofetil, 60 mg/kg/day in 3 doses	From day +5
Second HCT		
Conditioning regimen	Total body irradiation, 4 Gy, single fraction	Day −7
	Fludarabine, 35 mg/m^2^/day	From day −6 to day −3
	Cyclophosphamide, 30 mg/kg/day	From day −6 to day −3
	ATG Neovii^®^, 12.5 mg/kg/day	Days −2 and −3
GVHD prophylaxis	Mycophenolate mofetil, 60 mg/kg/day	From day +1
	Tacrolimus, 0.1 mg/kg intravenously, modulated according to blood levels	From day −1

ATG, anti-thymocyte globulin; GVHD, graft-versus-host disease; HCT, hematopoietic cell transplant.

**Table 2:** Antifungal susceptibility testing from parasternal abscess drainage (A) and cerebrospinal fluid (B) *.

MIC (A)	MIC (B)	Antifungal Drug
0.25	0.25	Voriconazole
0.5	1	Posaconazole
1	1	Itraconazole
1	2	Isavuconazole
8	8	Amphotericin
8	8	Micafungin

* CLSI interpretation not available.

**Table 3:** Summary of available evidence of scedosporiosis after HCT.

Reference	Outcome	Treatment	Organ Involvement	Age (Years)	N. of Cases
[10]	Mortality rate 68%	AmB/ICZ/VCZ	CNS 36% Pulmonary 40% Skin 38% Blood 25% Disseminated 69%	(mean +/− SD) 32.9 +/− 3.3	23 (of which 9 *L. prolificans*)
[11]	Died	AmB	CNS	34	1
[12]	Died	PCZ + AmB	Rhinoencephalitis	17	1
[13]	Survived	VCZ + AmB	Disseminated Pulmonary CNS	65	1
[15]	Survived	VCZ + AmB	Ocular Bone Heart CNS	59	1
[16]	Died	Intravitreal VCZ	Ocular CNS	58	1

AmB, amphotericin B; CNS, central nervous system; ICZ, itraconazole; PCZ, posaconazole; VCZ, voriconazole; SD, standard deviation.

**Table 4:** B-D-glucan test in cerebrospinal fluid (Wako turbidimetric assay).

BDG (pg/mL, Pos > 7)	Days Post-2nd HCT
7.35	114
3	126
173	172
26.4	189

## Data Availability

The original contributions presented in this study are included in the article. Further inquiries can be directed to the corresponding author.

## Abbreviations

The following abbreviations are used in this manuscript:

AML	acute myeloid leukemia
CGD	chronic granulomatous disease
GF	graft failure
GTX	granulocyte transfusion
GVHD	graft-versus-host disease
HCT	hematopoietic cell transplant
S.	scedosporium
TDM	Therapeutic Drug Monitoring
